# Mapping the Schizophrenia Genes by Neuroimaging: The Opportunities and the Challenges

**DOI:** 10.3390/ijms19010219

**Published:** 2018-01-11

**Authors:** Ayla Arslan

**Affiliations:** 1 Genetics and Bioengineering Program, Faculty of Engineering and Natural Sciences, International University of Sarajevo, Hrasnica cesta, 15 Ilidza, Sarajevo 71210, Bosnia and Herzegovina; aarslan@ius.edu.ba; 2 Department of Molecular Biology and Genetics, Faculty of Engineering and Natural Sciences, Uskudar University, Istanbul 34662, Turkey

**Keywords:** neuroimaging, brain, MRI, fMRI, GWAS, schizophrenia, magnetic resonance imaging, prefrontal cortex, common variants, single nucleotide polymorphism, imaging genomics, imaging genetics

## Abstract

Schizophrenia (SZ) is a heritable brain disease originating from a complex interaction of genetic and environmental factors. The genes underpinning the neurobiology of SZ are largely unknown but recent data suggest strong evidence for genetic variations, such as single nucleotide polymorphisms, making the brain vulnerable to the risk of SZ. Structural and functional brain mapping of these genetic variations are essential for the development of agents and tools for better diagnosis, treatment and prevention of SZ. Addressing this, neuroimaging methods in combination with genetic analysis have been increasingly used for almost 20 years. So-called imaging genetics, the opportunities of this approach along with its limitations for SZ research will be outlined in this invited paper. While the problems such as reproducibility, genetic effect size, specificity and sensitivity exist, opportunities such as multivariate analysis, development of multisite consortia for large-scale data collection, emergence of non-candidate gene (hypothesis-free) approach of neuroimaging genetics are likely to contribute to a rapid progress for gene discovery besides to gene validation studies that are related to SZ.

## 1. Introduction

Genetic variation, the differences in the DNA of a given species, is a key to fitness and survival. Increasing the chance of adaptability, the benefit of genetic variation may have come at a cost, however, which might be the case for schizophrenia (SZ). As a brain disease of disorganized thought and behavior, SZ is a complex disorder, which is caused by both genetic and environmental factors as well as their interactions. Genetic variations in human accelerated regions or HARs, for example, have been suggested as associated with SZ. HARs are short, human specific DNA segments, selected by natural selection [1,2]. Mostly involved in the regulation of gene expression and highly conserved across the span of vertebrate evolution, HARs seem to be rapidly evolved during hominization probably with some benefits specific to our species such as intellectual capacity. On the other hand, the mutations that are observed in HARs might have also prompted the risk of SZ, it turned out [3]. SZ is associated with alteration of prefrontal cortex (PFC) function, a brain region that is involved in higher cognitive processes. One of the regulator of PFC is the GABAergic signaling, the major inhibitory signal in the brain [4]. The PFC of the schizophrenic brain seems to lack a proper GABAergic function distorting the generation of gamma oscillations, leading to cognitive symptoms and deficits that are present in SZ [3]. But, what kind of variation in HARs specifically and in the entire human genome generally might be associated with the risk of SZ, exactly? 

Mapping *SZ* genes is not an easy task. First, genes do not make people schizophrenic. Rather, one form of genetic variation, i.e., single nucleotide polymorphism (SNP), which is naturally found in healthy human populations, is more common among the patients of SZ, thus, associated with the increased risk of SZ. Besides, this genetic risk has a polygenic nature [5], which makes the identification of SZ associated genetic variation a very complex task. Moreover, among the identical twins with such increased genetic risk, if one becomes schizophrenic, the risk to the other is on average less than 50%. Thus, environmental factors are also involved in the pathophysiology of SZ. Besides, mapping *SZ* genes require a usable genotype phenotype relationship however; SZ phenotypes are often clinical signs and symptoms, such as delusions, which are not specific, objective, and measurable for a precise and accurate analysis. Considering this huge complexity, hunting the SZ associated genes and associated pathways, and circuitries for better diagnosis, treatment and prevention have not been very fruitful so far [6].

### 1.1. Imaging Genetics

**Figure 1:**
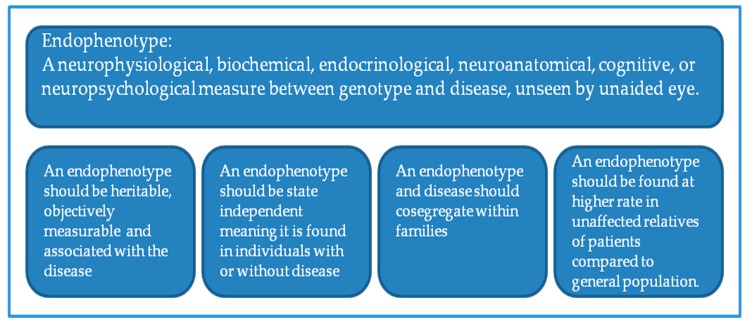
Definition of endophenotype and minimal selection criteria for psychiatric use.

One way to unmask the complexity of SZ pathophysiology would be perhaps to attack the problem on the site of phenotypes. Addressing this, one strategy would be the replacement of phenotypes with endophenotypes or intermediate phenotypes and linking them with genetic variations. In this context, neuroimaging parameters such as cortical thickness or function of PFC may be used as endophenotypes (Figure 1). Proposed to assist genetic analysis of complex traits, an endophenotype is a heritable character that co-segregates with a psychiatric illness, yet be present when the disease is not present, and be found in non-affected family members at a higher rate than in the population [7]. Thus, the use of endophenotype may potentially reduce genetic complexity and increase genetic effect size [8,9] for a better analysis of genetic risk factors of SZ, despite some concerns [6].

**Figure 2:**
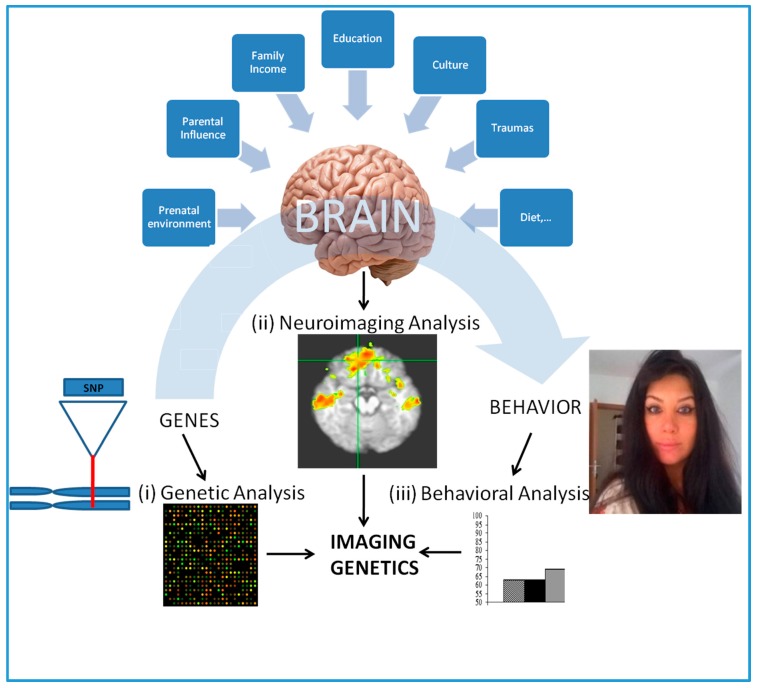
An overview of imaging genetics (IG). (**i**) Genetic analysis aims to detect the degree of specific or genome wide genetic variation (e.g., SNPs) among healthy or healthy/diseased subject groups; (**ii**) Neuroimaging analysis involves the study of specific or whole brain structural or functional brain endophenotypes of the subjects by in vivo neuroimaging. This could be further explored in the context of environmental insults; (**iii**) Behavioral analysis involves the study of behavioral patterns of the subjects and incorporated (if available) in to genetic and neuroimaging data to test for any statistically significant association.

Consequently, incorporating neuroimaging endophenotypes with the genetic data from healthy and/or diseased subjects, the effect genetic variation, such as SNPs, on the brain structure and function can be studied [6,10,11,12,13]. So-called imaging genetics (IG), this methodology typically requires the utilization of endophenotypes derived from neuroimaging data to test the genetic association of a relatively well validated SZ candidate gene (this is called as “candidate gene approach” [6]). Figure 2 presents an overview of this methodology, which is performed by analysis in three different levels of complexity, genetic level, neurological level and behavioral level. Hence, the methodology explores how does the effect of a set of genetic variance, which maybe greater among SZ patients when compared to healthy subjects contribute to structural and/or functional brain pathogenesis or making the relevant brain regions more susceptible to environmental insults. Although the environmental insults specific for SZ have yet to be examined, several studies have recently used IG to study gene-environment interactions as a mechanism for psychopathology including attention-deficit/hyperactivity disorder (ADHD) [14]. 

Early IG studies, which emerged in the beginning of 2000s, typically used a so-called “candidate gene approach”, focusing on a single genetic variation such as a functionally relevant SNP found in relatively well validated single gene where as more recent studies used a wider range of genetic data or genome wide scans (this latter is called as non-candidate gene approach) [6,15]. The genetic data are compared with neuroimaging data that involve the structural or functional brain endophenotypes. These endophenotypes are either a relatively well validated candidate phenotype such as volume of hippocampus or connectivity of hippocampus with dorsolateral prefrontal cortex (DLPFC) or brain-wide phenotypes such as whole brain white matter integrity or global grey matter density [6]. Also, it is important to note that, endophenotypes used for IG are sometimes called as intermediate phenotypes [6,10] as endophenotypes were originally proposed for psychiatric gene discovery by Irving Gottesman, a pioneer of SZ genetics, who passed away in 29 June 2016. 

As tools for genetic and neuroimaging analysis accumulate, the last twenty years have witnessed an increasing IG analysis of SZ, which has yielded numerous publications but even the robust classical genetic candidates like the gene encoding for the enzyme Catechol-*O*-methyltransferase (COMT) has yet to be confirmed [16,17]. These initial studies of IG have adapted the “candidate gene approach”, which, used the “historical SZ candidate genes” for an association with a given endophenotype. These genes were typically identified by genetic linkage [18] and association studies [19]. The latter approach seems more powerful than the former. For example, the *SZ* Gene database (http://www.szgene.org/) show that there are 1727 association studies, leading to the identification of 1008 genes, 8788 SNPs (Last updated in 23 December 2011). Thus, relevant literature produced a list of SNPs found in the “historical SZ candidate genes”, such as *COMT* gene-involved in the degradation of catecholamines, including dopamine [20,21]. However, these findings were not successful to clearly explain the genetic basis of SZ [17]. Moreover, neuroimaging data have been a target of major discussion for concerns about its specificity [22,23,24], although this field is under rapid progress [25].

### 1.2. A Controversial SZ Candidate Gene Encoding for Catechol-O-methyltransferase (COMT) and Others

The first studies of IG studies focused on the available “historical candidate genes” of pre-GWAS era, as described above. Thus, a relatively well known genetic variation has been tested for neuroimaging endophenotypes, such as structure or function of hippocampus [26,27]. 

Among these SZ candidate genes, the *COMT* gene has been one of the most exploited molecular targets of SZ. It is located in the human chromosome 22, which has been known to be enriched by *SZ* genes according to genetic studies [28,29,30]. A deletion in this region (1/4000 human births) increases the likelihood of developing SZ about 25 times [31]. So far, most SZ studies have focused on the *COMT* gene variation that causes the amino acid valine (Val) at position 108 to be replaced with methionine (Met). This variation leads to a 3- to 4-fold decrease in the thermo-stability of the gene product, the COMT enzyme, which in turn alter the dopamine levels. 

Consequently, many studies, using the “candidate gene approach” targeted the *COMT* gene to validate its association with SZ [32,33,34,35,36]. For example, the ground breaking IG study of Egan et al. (2001) [32] suggested a relation between COMT Val108/158 Met genotype, frontal lobe function and risk for SZ. The study is also one of the frontiers in the candidate gene studies of IG in the SZ research as it uses a form of measurable brain phenotype called an endophenotype as determined by functional magnetic resonance imaging (fMRI) and linking it with genetic variation. Thus, a relatively well validated candidate gene variation, such as COMT Val108/158 Met genotype, is further studied for SZ association using neuroimaging as a tool. However, the study requires further confirmation due to contradicting results [16].

Naturally, COMT alone is not sufficient to cause SZ, but still its significance could be better understood if it is investigated in the context of gene-gene and gene-environment interactions. Addressing this, one study for example, used functional magnetic resonance imaging (fMRI) to analyze effect of DNA methylation in the membrane bound-COMT promoter by measuring the neural activity in the DLPFC during working memory [37]. Thus, the story of COMT has not finished, yet.

Other candidate gene studies (please also see [6] for a comprehensive review of candidate gene studies of pre-GWAS) include the effect of genetic variation on the gene encoding for Brain derived neurotrophic factor (BDNF)—a neuroptophin involved in neuro-development, neuro-protection, synaptic plasticity, learning, and memory [38,39], Dysbindin (DTNBP1)—a protein that may play a role in organelle biogenesis [40,41]. But results were not very convincing. For example, a meta-analysis, testing the IG studies for association between functionally relevant Val66Met polymorphism in the *BDNF* gene and hippocampal volumes has failed to validate a significant association [42].

## 2. The New Age

In the meanwhile, new molecular tools, such as SNP maps, have catalyzed the genome-wide association studies (GWAS), offering a better strategy for disentanglement of the enormous genetic complexity of SZ. More than 30 GWAS have been conducted for SZ research until now [43]. The SNPs resulted from these studies have been mapped to genomic regions relevant to pathways of glutamatergic and dopaminergic systems, as well as calcium signaling and inflammatory mechanisms, which altogether open new avenues for SZ research [44]. Consequently, these findings are the major theme of molecular, cellular, physiological and behavioral studies both in humans and animals [45,46,47,48]. These efforts have been supported by some recent meta-analysis. Results show that studies investigating factors related to the immune system in post-mortem brains of SZ patients and healthy controls have supported the previous findings [49]. 

As more and more studies address the genetic variations that are related to psychiatric disorders in general and related to SZ in particular, analysis of the effect of these genetic variations produced by each SNP alone or in combination at the level of neural circuitries and linking them with behavior is essential [6]. This means more opportunities for IG. Emergence of new candidate genes of SZ naturally catalyzes their validation studies by IG. This part and its major pitfalls are presented in the below section called “Novel SZ candidate genes: The cherry on the cake”.

Moreover, the elusive problems such as reproducibility, genetic effect size, genetic validity and sensitivity of neuroimaging endophenotypes, are slowly addressed by some new progress such as multi site consortia or multivariate analysis methodologies. Thus, IG has also become instrumental for the discovery of new candidate genes by hypothesis free approaches (“non-candidate gene strategy” [6]. This part is described under the section called: “Large scale multivariate data: The low hanging fruit”.

### 2.1. Novel SZ Candidate Genes: The Cherry on the Cake

**Figure 3:**
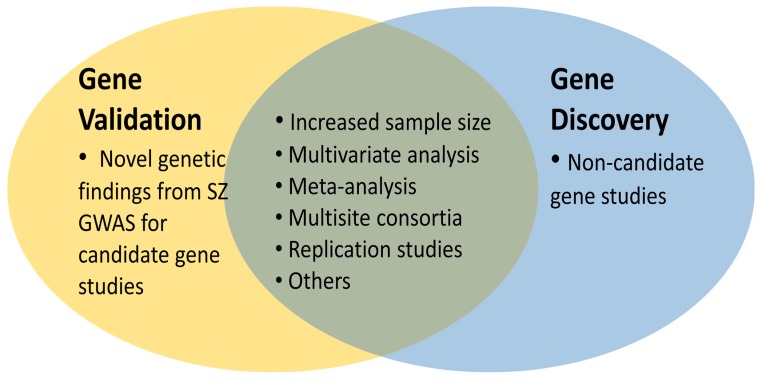
The opportunities for imaging genetic analysis of SZ. Imaging genetics can be used for gene validation and gene discovery, both of which will certainly benefit from increased sample size, multivariate analysis, meta-analysis, multisite consortia, replication studies and many others such as progress in neuroimaging technology. (SZ: Schizophrenia, GWAS: Genome wide association studies).

GWAS continuously produce new data more than ever but they fail to validate previous “historical candidate genes” for SZ but rather novel ones. Several GWAS suggest variations found in more than 100 loci associated with SZ risk, but they did not confirm previously popular candidate *SZ* genes [50,51,52,53,54,55,56,57,58,59,60,61,62,63]. Gradually, these novel genetic findings have been studied as “new candidate genes” of IG. Thus, these new genetic findings are new source of candidate gene studies of imaging genetics for gene validation (Figure 3).

Among the common SNP variants identified from the recent GWAS for example, variations in CACNA1C have been implicated as calcium signaling SNPs, found to confer the risk for schizophrenia [62]. Addressing this, several studies analyzed the calcium voltage-gated channel subunit α1 C-gene (CACNA1C) variations by the use of electrophysiological endophenotypes assessing phases of sleep in infants [64] by measuring regional gray matter volume [65], by measuring amygdale structure and/or function [66,67] by proton magnetic resonance spectroscopy of glutamatergic signals [68]. In the meanwhile, animal studies generate conditional knock-out mice for the analysis of calcium signaling in cellular level, linked to neuropsychiatric diseases [69,70].

Another recently identified genome-wide significant schizophrenia genetic risk variant is the major histocompatibility (MHC) locus, which was tested for associations on cognition and brain structure in 346 patients of SZ and 2342 healthy comparison subjects [71]. The MHC locus is one of the most significant determinants of SZ susceptibility [51]. However, it is hard to precisely map the relevant SNPs, as the majority of the SNPs are likely located in the non-coding genomic regions that cannot be easily correlated to functional significance to explain the risk of SZ [72]. Indeed, there is a trend of SNPs with genome wide significance, which falls into non-coding region of genomic DNA thus, directing imaging genetics towards imaging genomics besides to imaging epigenomics. For example, imaging genetic studies start to decode epigenetic elements involved in the macro- and microstructure of the white matter of the human brain [73]. 

Despite these powerful GWAS, the SNPs identified so far were not able to explain the sources of genetic variation associated with SZ, completely. Some genetic variants, such as common variants of very weak effect, low-frequency, and rare variants of small to modest effect, or a combination of both could be possibly not captured by these association studies leading to the phenomenon of “missing heritability” [74,75]. Regarding this, new genetic candidates are being continuously reported including copy number variations, which were hard to capture by previous GWAS [76,77]. While some studies analyze the CNV effect for relevant endophenotypes for SZ associations [78] more studies are required. 

Indeed, SZ GWAS variants have not yet sufficiently been explored by imaging genetics, thus, more studies are expected in this field soon. As shown in the Figure 3, this will be particularly fruitful for gene validation in the context of increased sample size, multivariate analysis, multisite consortia such as ENIGMA consortium [79], which will be important for meta-analysis. Also, independent replication studies are required. Consequently, novel SZ candidate genes with genome wide significance appear to be the “cherry on the cake” for the candidate gene studies of IG that were previously constrained by “historical SZ candidate genes” with limited validity and genetic effect sizes. Thus, there is a growing opportunity for better exploration in the field.

### 2.2. Large Scale Multivariate Data: The Low Hanging Fruits

Indeed, IG studies have always been challenged by small sample size, lack of standard study designs, and statistical power [15]. These in turn caused poor results of meta-analysis and inconsistent results. In this context, the development of consortia and large-scale data collection platforms seem to be particularly significant along with multivariate data analysis [15]. GWAS continuously decode SZ risk genes [43]. Global neuroimaging consortia produce collections of brain scans from tens of thousands of people [80]. Along with these, a non-candidate gene approach that analyses the whole genome data with structural and functional brain endophenotype(s), has also been adapted. As presented previously by Arslan [6] there are different versions of this strategy such as whole genome is tested for association with one specific brain phenotype or brain wide phenotype. These studies are particularly essential for gene discovery (Figure 3). For example, a recent GWAS of 33,536 individuals has used this non-candidate gene strategy which led to the identification of six loci significantly associated with hippocampal volume [81]. These loci include rs77956314 and rs61921502 at chromosome 12 and both SNPs are found in the non-coding regions: The former is found in an intergenic region located in the 4 kb upstream of *HRK* gene- the gene encoding a member of the BCL-2 protein family and involved in apoptosis(provided by RefSeq, October 2012)—whereas, the latter is found in the intron of MSRB3—the gene encoding a protein which catalyzes the reduction of methionine sulfoxide to methionine (provided by RefSeq, July 2010) as well as rs11979341 in chromosome 7 in a region 200 kb upstream of SHH gene, which encodes a protein implicated as the key inductive signal in patterning of the ventral neural tube, the anterior-posterior limb axis, and the ventral somites (Entrez Gene ID: 6469).The other loci are rs7020341, rs2268894, rs228988, located in the introns of ASTN2—a SZ associated gene encoding for a protein that may be involved in neuronal migration (provided by RefSeq, May 2010) DPP4—a gene encoding membrane glycoprotein (provided by RefSeq, July 2008)—and MAST4—a gene a encoding a microtubule-associated serine/threonine protein kinase (provided by RefSeq, March 2014)—in the chromosomes 9, 2, 5, respectively. 

Another recent study has reported the effect genetic variation on the prenatal brain development by a combined analysis of GWAS of global brain tissue volumes in 561 infants. Results show that rs114518130 was significantly associated with gray matter volume (*p* = 4.15 × 10^−10^), which was not predicted by rare copy number variants or genetic predisposition scores for SZ [82]. Detection of the repertoire of variants impacting on developmental pathways of SZ is particularly significant for disease prevention. Thus, this stream of IG is essential.

The recent progress in IG in the direction of non-candidate gene approach (Figure 3), which tests relations between large scale genomic data and millions of brain signals, has catalyzed the progress in multivariate pattern analysis methods, also [15]. As the problems of specificity and sensitivity have been commonly addressed for neuroimaging data [22] this seems to be particularly beneficial for reliable results [83]. For example, it was shown that multivariate analysis of brain functional and structural alterations differentiates schizophrenic patients from healthy controls with 80% sensitivity and specificity [84]. Thus, if one hanging fruit of IG is the gene discovery, the others will come by the use of large-scale multivariate analysis [85] with progressive methodologies [86]. Indeed, a recent publication already shows the significance of the multivariate techniques addressing the relations between brain structures, cognitive and polygenetic risk score in the classification of patient groups [87].

However, multivariate analysis requires preliminary characterization of validated shared genetic relations between variants. For example, one study [88] analyzed the amount of shared genetic variance between the total volume of hippocampus and volume of sub-regions of hippocampus and episodic memory performance in a sample size of 499 siblings (mean age ± SD = 30.0 ± 3.1, 203 men), including 51 monozygotic and 46 dizygotic twin pairs and 305 non-twin siblings, collected by the Human Connectome Project. Data show that the amount of shared genetic variance between sub-regions of hippocampus and verbal episodic memory was low (mean = 0.10, range = 0.01–0.20). Thus, these two traits seem not to be suitable for bi-variate analysis, for example. 

## 3. Conclusions

IG studies of neuropsychiatric disorders including SZ have been increasingly published in the literature for almost twenty years. These years have witnessed the problems of reproducibility, genetic effect size, genetic validity, specificity, and sensitivity of neuroimaging endophenotypes. For example, neuroimaging has been a target of major discussion for concerns about its specificity [22,23,24].

On the other hand, neuroimaging methodology is under rapid progress with the advent of imaging at ultrahigh fields [25]. Besides, neuroimaging data will likely get a boost with multivariate analysis [84], so, the use of neuroimaging as endophenotypes will likely to grow.

Moreover, the recent progress in GWAS has resulted in renewed optimism to understand the genetic architecture of SZ. GWAS have identified a number of common polymorphisms, such as MHC locus, which is strongly associated with SZ, besides to microRNAs (miRNAs) (reviewed in [89]), or rare variants, such as CNVs [76,77], for example. These findings require further analysis and IG is one way to address their validation. This trend will also guide other studies for better understanding and dissection of SZ relevant circuitry in animal models by optogenetics, for example [90,91]. Moreover, as miRNAs, are affected by epigenetic mechanisms such as DNA methylation, incorporation of relevant imaging, genetic, and epigenetic data will be important to elucidate the mechanisms of gene-gene and gene-environment interactions, which may be associated with SZ endophenotypes [92].

In the meanwhile, there have been better platforms, tools and methods for the analysis of large scale multivariate data, and a rapid increase in the multisite studies [93,94] for systematic, large scale collection of genomic and phenomic data. These caused progresses in the design of IG studies such as “non-candidate gene approach” [95] for gene discovery in addition to “candidate gene studies” (hypothesis-driven approach). These approaches will increasingly benefit from the trend of increased sample size, multivariate analysis, and multisite consortia. Besides, there is a space in the field for more replication studies and meta-analysis (Figure 3). Consequently, in the light of current progress, IG research holds a strong translational and integrative potential for better diagnosis, treatment, and prevention of SZ.

## Abbreviations

ADHD	Attention deficit hyperactivity disorder
BDNF	Brain derived neurotrophic factor
CACNA1C	Calcium voltage-gated channel subunit α1 C-gene
CNV	Copy number variation
COMT	Catechol-*O*-methyltransferase
DLPFC	Dorsolateral prefrontal cortex
fMRI	Functional magnetic resonance imaging
GWAS	Genome wide association studies
IG	Imaging genetics
miRNAs	Micro RNA
MHC	Major histocompatibility complex
MRI	Magnetic resonance imaging
PGC	Psychiatric genetics consortium
SZ	Schizophrenia
